# Chronic Tophaceous Gout Presenting With Severe Polyarticular Erosive Disease: A Case Report

**DOI:** 10.7759/cureus.101502

**Published:** 2026-01-14

**Authors:** Ana Rita Ambrósio, Telma Costa Cabral, Laura Gago, Hugo Pêgo, Isménia Oliveira

**Affiliations:** 1 Internal Medicine, Hospital Beatriz Ângelo, Loures, PRT; 2 Rheumatology, Hospital Beatriz Ângelo, Loures, PRT

**Keywords:** extensive tophi, joint deformities, polyarticular gout, tophaceous gout, tophus drainage

## Abstract

Gout is an inflammatory disease caused by deposits of monosodium urate crystals in joints, bone, and surrounding soft tissues. It can present as acute gout flares, chronic gouty arthritis, and tophaceous gout. A 63-year-old man with long-standing hyperuricemia and tophaceous gout, intermittent adherence to therapy, and alcohol use presented with polyarticular pain and swelling during hospitalization for an infected venous leg ulcer. Physical examination showed multiple tophi and deformities of the metacarpophalangeal and interphalangeal joints, spontaneous drainage of white material from the right elbow, and swollen knees with exudation from the left knee. Serum uric acid was 4.6 mg/dL (reference range 3.4-7.0). Radiographs demonstrated hand erosions and hook-shaped osteophytes. The right elbow had punched-out erosions with periarticular soft-tissue densification and bilateral gonarthrosis with fibular-head erosions. He was treated with corticosteroids, colchicine, and febuxostat, with clinical improvement, and remains under outpatient follow-up. Tophaceous gout can lead to extensive joint and soft-tissue destruction despite normal serum-urate levels; clinical history and imaging are essential to assess disease burden and guide management.

## Introduction

Gout is a common crystal-induced arthropathy with increasing worldwide prevalence and is more common in men than in women. It results from deposition of monosodium urate crystals in articular and non-articular tissues [1-4].

Clinically, it is often described as a continuum. Acute flares most often involve the first metatarsophalangeal joint (podagra), a finding considered a hallmark of gout. However, virtually any joint may be affected, and urate deposition can extend to periarticular and extra-articular tissues. Patients typically experience intermittent episodes of abrupt, intensely painful inflammatory arthritis that are self-limited, typically resolving within one to two weeks (7-14 days), with asymptomatic intervals between flares. These episodes reflect an innate immune response to pre-existing monosodium urate deposits and are frequently precipitated by factors such as medication changes, alcohol intake, intercurrent illness, hospitalization, or surgery [1-4].

Persistently elevated serum urate is the key driver of monosodium urate crystal formation. The risk of crystal deposition rises when serum urate is persistently high, although it may test within the normal range during acute flares. When serum urate is not adequately controlled over time, gout often progresses from isolated attacks to chronic tophaceous disease, characterized by more frequent and polyarticular flares, persistent joint pain and stiffness, and substantial crystal deposition in joints and soft tissues [1-3].

Gout is recognized as a chronic disease associated with increased cardiovascular morbidity and mortality [1-4]. Cardiometabolic comorbidities are prevalent in this population and are considered part of the typical comorbidity profile of gout [1-6]. Accordingly, contemporary guidance recommends systematic screening and active management of cardiovascular and metabolic risk factors at diagnosis and throughout follow-up as an integral component of gout care [3-6].

Long-term urate-lowering therapy is central to gout management, as sustained reduction of serum urate promotes dissolution of monosodium urate crystals and helps prevent future flares [3-7]. However, initiating or intensifying this therapy can temporarily increase flare risk, probably due to mobilization of urate stores [3-7]. At the same time, gout flares need to be treated quickly to ease pain and preserve function [1,4].

We present the case of a 63-year-old man with long-standing tophaceous gout who was admitted with infected venous leg ulcers and developed a severe tophaceous gout flare during the hospitalization. This case illustrates the complexity of managing advanced gout in a multimorbid patient with active infection and emphasizes the need to balance infection control, anti-inflammatory therapy, and optimized urate-lowering treatment within a coordinated multidisciplinary care framework.

## Case presentation

A 63-year-old retired man, previously independent, presented to the emergency department with several days of worsening pain in both lower limbs, associated with fever and progressive functional limitation. He reported a chronic exudative ulcer on the right leg and a wound over the left calcaneus, both under regular dressing care at his primary care center, noting that limb pain and difficulty walking had worsened since the last treatment, three days before admission.

His medical history included long-standing tophaceous gout with prior hospitalizations for acute flares and rheumatology follow-up (rheumatoid factor and anti-cyclic citrullinated peptide (CCP2) antibodies negative), hypertensive heart disease, dyslipidemia, obesity, colonic diverticulosis, alcohol-related chronic liver disease, chronic deep venous insufficiency with venous leg ulcers, severe bilateral gonarthrosis, a history of bilateral femoropopliteal deep vein thrombosis complicated by pulmonary embolism, bladder cancer treated surgically about 10 years earlier, and a surgically managed intestinal perforation. He denied smoking but reported regular consumption of wine and beer. Usual medication included rosuvastatin 20 mg, losartan 100 mg, folic acid 5 mg, and allopurinol 300 mg taken daily (occasionally self-escalated to twice daily during flares), and paracetamol, naproxen, and colchicine as needed.

At presentation, his blood pressure was 114/67 mmHg, heart rate 111 beats per minute, he was febrile with a temperature of 39°C, and oxygen saturation was 94% on room air. Physical examination showed multiple firm nodules compatible with tophi over both elbows and hands. Examination of the lower limbs revealed ulcers with a fibrinous base. There was no marked erythema, but both lower limbs were warm, with mild bilateral malleolar edema, diffuse tenderness to palpation, and chronic venous changes, including hyperpigmentation and lipodermatosclerosis. Initial laboratory findings are summarized in Table 1 and showed normocytic anemia (hemoglobin 11.6 g/dL), leukocytosis (15.33 × 10^9^/L; neutrophils 83.8%), a normal platelet count, markedly elevated C-reactive protein (CRP 224 mg/L), preserved renal function (estimated glomerular filtration rate 68 mL/min/1.73 m^2^), liver enzymes within the reference range at admission, and N-terminal pro-B-type natriuretic peptide (NT-proBNP) of 1309 pg/mL (Table 1).

**Table 1:** Laboratory findings at admission (with reference ranges). CRP: C-reactive protein; NT-proBNP: N-terminal pro-B-type natriuretic peptide

Parameters (unit)	Patient values (at admission)	Reference range
Hemoglobin (g/dL)	11.6	12-15
Leukocytes (x10^9^/L)	15.33	4.5-11
Neutrophils (%)	83.8	40-75
Platelets (x10^9^/L)	314	150-450
CRP (mg/L)	224	<5
Creatinine (mg/dL)	1.13	0.67-1.17
Urea (mg/dL)	21	16.6-48.5
Total Bilirrubin (mg/dL)	0.6	<1.4
Conjugated Bilirrubin (mg/dL)	0.23	<0.5
Aspartate Aminotransferase (U/L)	20	<40
Alanine Aminotransferase (U/L)	7	<41
Alkaline Phosphatase (U/L)	108	40-129
Gamma-Glutamyl Transferase (U/L)	24	<60
Lactate Dehydrogenase (U/L)	260	135-225
Sodium (mEq/L)	140	136-145
Potassium (mEq/L)	4.7	3.5-5.10
Chloride (mEq/L)	100	98-107
NT-proBNP (pg/mL)	1309	<386

He was admitted for infected venous leg ulcers, and blood and urine cultures were obtained before starting empirical flucloxacillin. Arterial duplex ultrasonography of the lower limbs showed preserved triphasic flow to the feet via the posterior tibial arteries, and venous duplex revealed no femoral or popliteal deep vein thrombosis bilaterally, findings consistent with chronic venous insufficiency with stasis ulcers.

Over the following days, fever and inflammatory markers worsened, with progression of anemia (hemoglobin 9.7 g/dL), persistent leukocytosis (14.45 × 10^9^/L with neutrophilia), mild renal impairment (creatinine 1.4 mg/dL), and CRP rising to 349 mg/L, prompting escalation of antibiotic therapy to piperacillin-tazobactam. He subsequently became afebrile, with progressive improvement in the appearance of the ulcers, and blood and urine cultures obtained at admission remained negative. His usual allopurinol was continued during hospitalization. During this period, he developed marked worsening of joint pain and functional limitation, with severe pain in the right elbow, multiple metacarpophalangeal and interphalangeal joints, both knees, and both ankles, resulting in major impairment of mobility and self-care.

**Figure 1:**
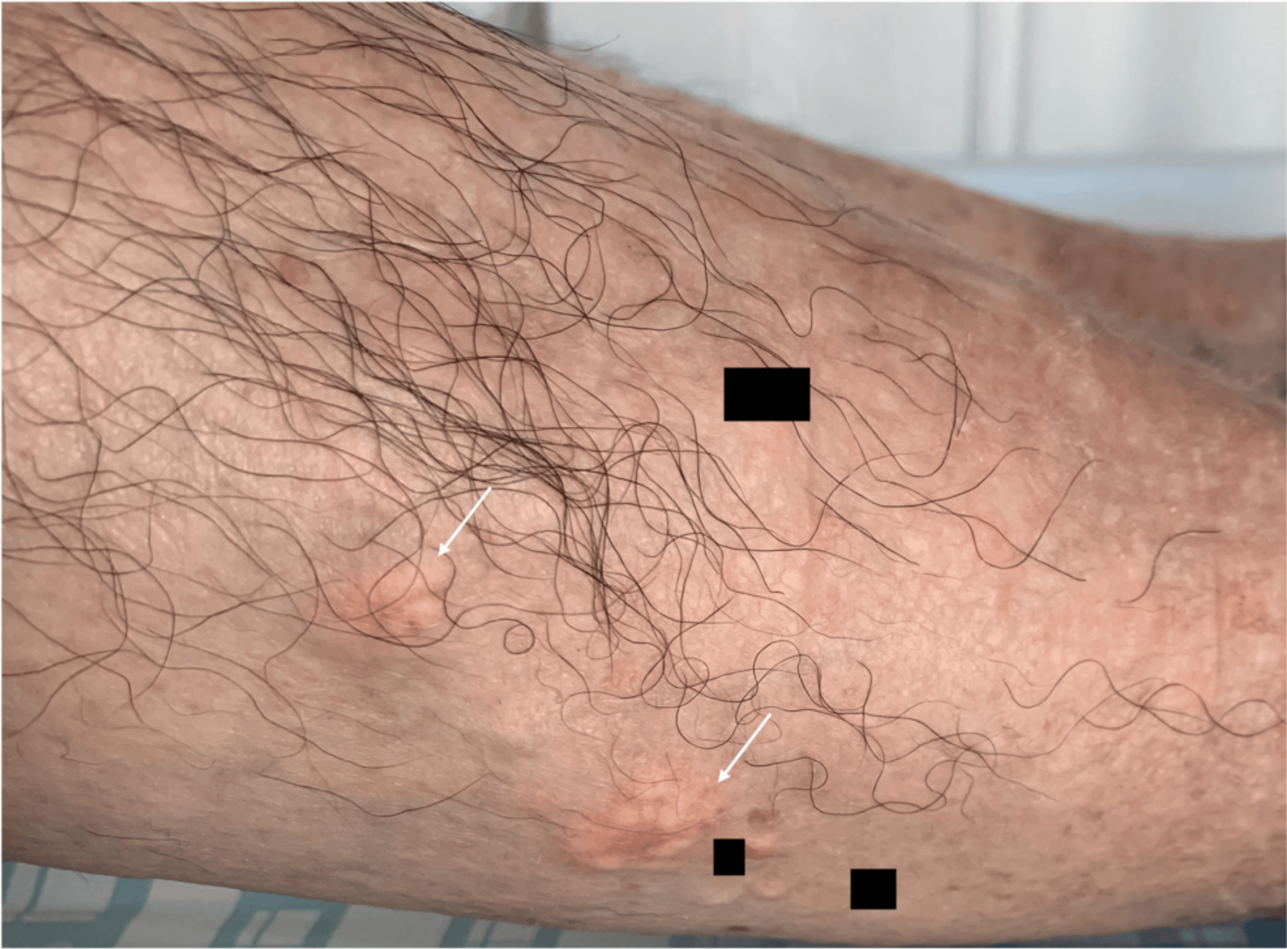
Subcutaneous tophi over the posterior aspect of the right calf (arrows) in a patient with long-standing tophaceous gout.

**Figure 2:**
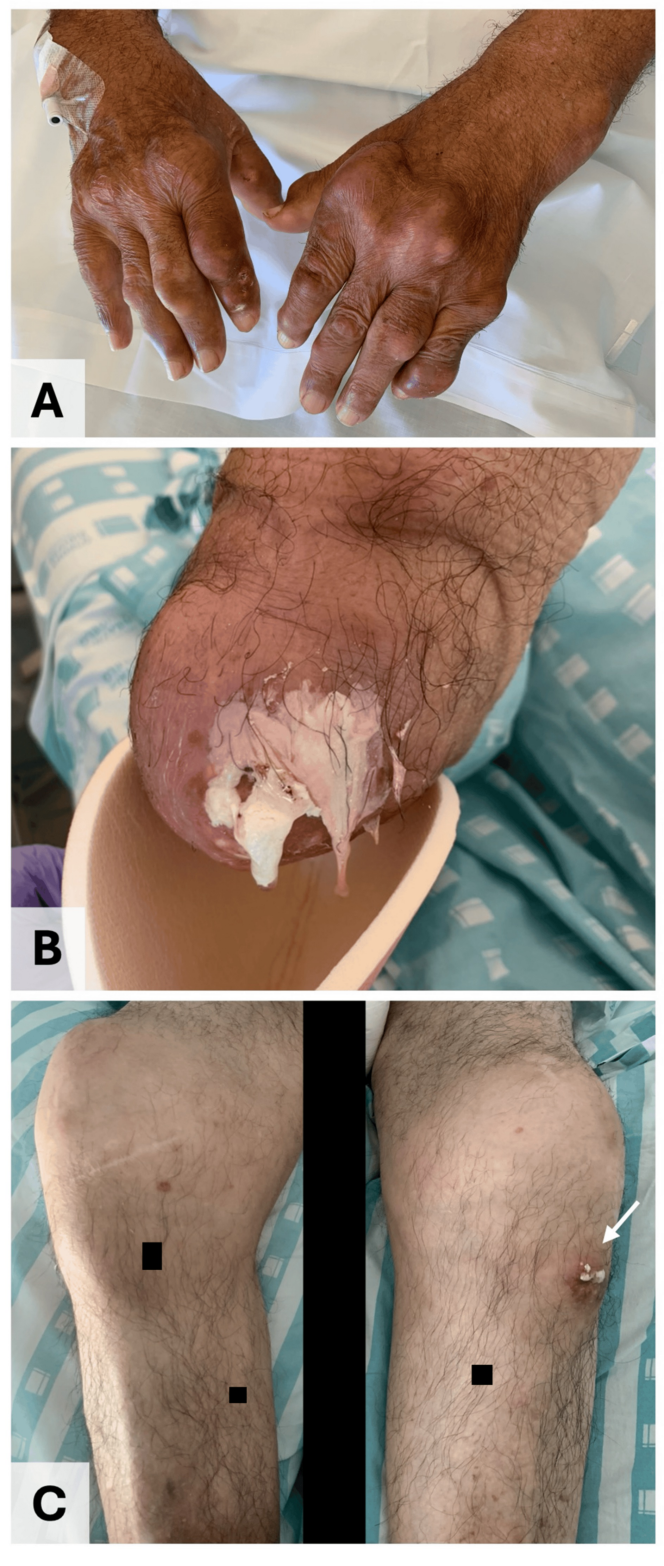
Clinical manifestations of tophaceous gout. (A) Multiple tophi deforming the metacarpophalangeal and interphalangeal joints of both hands. (B) Right elbow with spontaneous drainage of thick, white, paste-like tophaceous material. (C) Swollen knees with tophaceous exudate at the left knee (arrow).

**Figure 3:**
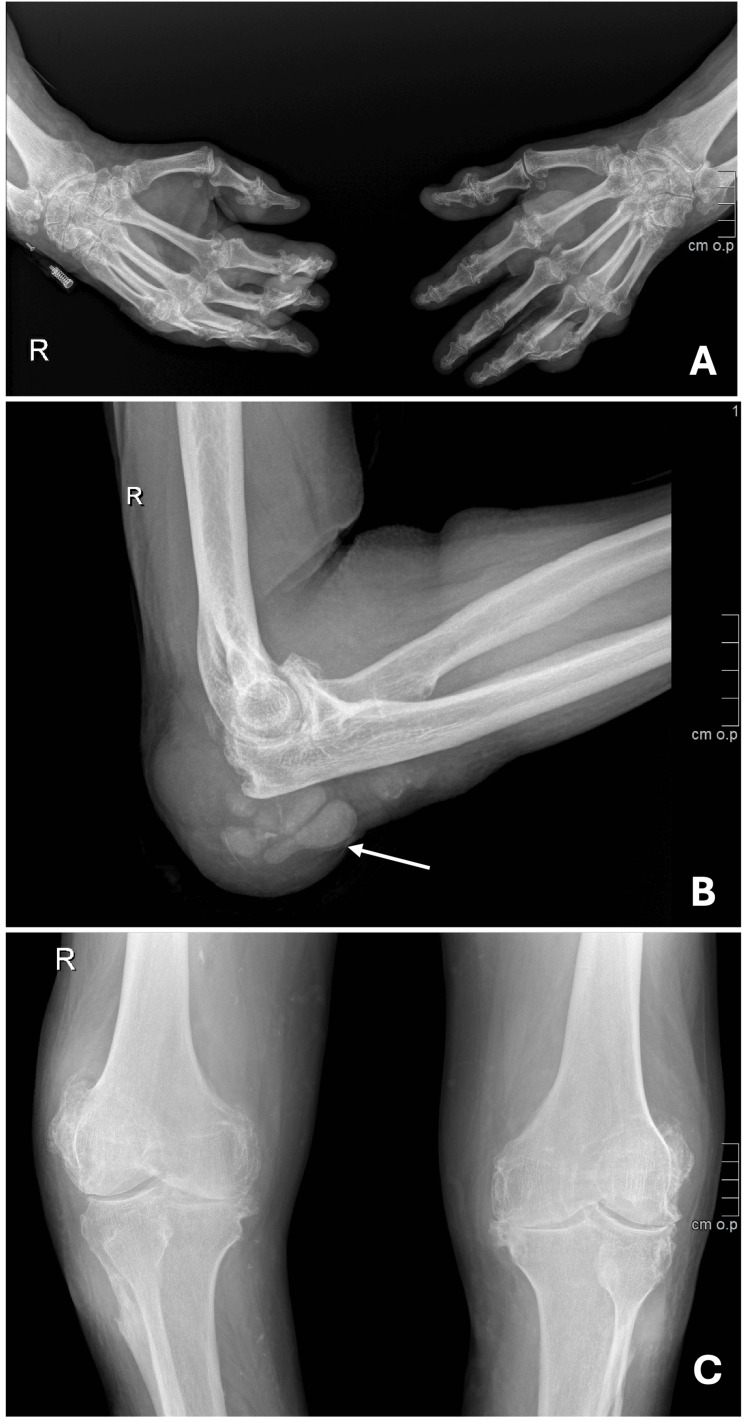
Radiographic features of tophaceous gout (A) Hand radiograph showing multiple erosions and prominent osteophytes. (B) Right elbow radiograph demonstrating punched-out periarticular erosions with overhanging edges and increased periarticular soft-tissue density compatible with tophi (arrow). (C) Bilateral knee radiographs showing severe knee osteoarthritis with large hook-like osteophytes and erosions of the fibular head.

Examination revealed polyarticular inflammatory arthritis characterized by swollen, warm, tender joints and subcutaneous tophi over the calves (Figure 1). Multiple tophi were also deforming the metacarpophalangeal and interphalangeal joints of the hands (Figure 2A) and feet. There was spontaneous drainage of thick, white, paste-like material consistent with tophaceous debris from the right elbow (Figure 2B), the left knee (arrow, Figure 2C), the distal interphalangeal joint of the left fifth finger, and the first and second fingers of the right hand. Left olecranon bursitis was also noted. Serum uric acid was 4.6 mg/dL (reference range 3.4-7.0). Hand radiographs revealed multiple erosions and prominent osteophytes (Figure 3A). Radiographs of the right elbow showed typical “punched-out” periarticular erosions with overhanging edges and increased periarticular soft-tissue density compatible with tophi (arrow, Figure 3B), and knee radiographs demonstrated severe bilateral knee osteoarthritis with large hook-like osteophytes and erosions of the fibular head (Figure 3C). He was evaluated by general surgery and plastic surgery for wound and soft-tissue management, with no indication for acute surgical intervention.

Colchicine was initiated for the acute flare but required dose reduction due to diarrhea. Systemic glucocorticoid therapy with prednisolone 20 mg daily was associated with gradual improvement in joint pain and mobility, and in view of the severity of the flare and persistent active arthritis, treatment was continued beyond five days, with the dose maintained for nine days and then tapered. Calcium and vitamin D supplementation were introduced. The rheumatology team was involved and, given the severity of tophaceous disease and suboptimal control on allopurinol, urate-lowering therapy was switched to febuxostat during hospitalization, without immediate adverse events, with plans for outpatient titration and monitoring.

The physical medicine and rehabilitation team implemented a tailored program focusing on joint mobility, strengthening, gait training, and transfers. As the infection was controlled and the gout flare subsided, the patient regained partial independence, although significant limitations from chronic joint damage and gonarthrosis persisted. He was referred to a rehabilitation unit for intensive motor rehabilitation to prevent further functional decline and help recover function, with continued outpatient follow-up in rheumatology, orthopedics, and plastic surgery.

## Discussion

Gout is a chronic crystal-induced arthritis that, when serum urate is not adequately controlled, may evolve from intermittent flares to chronic tophaceous, polyarticular disease with substantial disability [1-4,8,9]. In the present case, gout had progressed to an advanced tophaceous stage, with severe deforming polyarthritis, extensive subcutaneous and articular tophi, and major functional impairment. The coexistence of cardiovascular disease, chronic liver disease, venous insufficiency with leg ulcers, and obesity reflects the strong association between gout and cardiometabolic and vascular comorbidities. It also highlights that, even in the acute setting, these associated risk factors should be actively optimized and managed in parallel with gout-directed therapy [1-4,9].

Despite the presence of widespread tophi and classic radiographic damage, serum uric acid was within the reference range during the acute flare. This finding underscores the limited diagnostic value of a serum urate measurement obtained during an acute attack or intercurrent illness, as hyperuricemia may be absent and levels may even be within the normal range, probably due to increased renal urate excretion during the acute-phase response [1-3,8,9].

In this patient, the severity of tophaceous disease most likely reflects many years of inadequately controlled hyperuricemia in the context of multimorbidity, contributing to a sustained crystal burden and progressive joint damage [1,4,9]. His regular consumption of wine and beer is also relevant, as observational data indicate that alcohol-related gout risk is dose- and beverage-dependent, with beer conferring a greater increase in risk than wine [10]. Emerging evidence further suggests that inter-individual susceptibility to gout, and the impact of environmental and lifestyle factors more broadly, is modulated by genetic variation in pathways involved in urate handling and inflammation, highlighting the increasingly recognized role of genetic background in shaping gout risk [2,3,9].

The pattern of joint involvement in this patient, with prominent small-joint disease of the hands, deforming tophi, and characteristic erosions, aligns with published descriptions of chronic hand involvement in gout and can mimic other forms of inflammatory arthritis, such as rheumatoid arthritis [2,3,8,11,12]. In this setting, imaging helps define the extent of joint involvement, with radiographs demonstrating typical “punched-out” erosions with overhanging edges and periarticular soft-tissue masses, consistent with long-standing urate deposition [2,3,7,11]. Although advanced modalities such as ultrasound and dual-energy CT are increasingly recommended to detect urate deposits and monitor response to therapy, conventional radiography remains an informative and readily accessible tool in advanced disease [3,7,9].

Treatment was particularly challenging because of active soft-tissue infection and multiple comorbidities. Current guidelines support the use of colchicine, nonsteroidal anti-inflammatory drugs (NSAIDs), or glucocorticoids for acute flares, with the choice guided by comorbidities and contraindications [1,4,5,8]. In this patient, NSAIDs were avoided because of cardiovascular and renal disease, and low-dose colchicine required reduction due to gastrointestinal intolerance, so systemic glucocorticoids were used despite infection concerns, alongside optimized antibiotic therapy and close monitoring [1,4,5]. For long-term control, a treat-to-target urate-lowering approach is recommended, usually starting with dose-titrated allopurinol, with febuxostat, a xanthine oxidase inhibitor, considered when urate targets are not reached, or allopurinol is not suitable [3,5,6,9]. In this case, escalation to febuxostat was appropriate given the severity of tophaceous disease and suboptimal urate control on a fixed-dose allopurinol regimen, likely influenced by intermittent adherence [3,5,6,9].

Finally, this case illustrates that advanced tophaceous gout can lead to marked functional impairment and a prolonged recovery, reinforcing the need for individualized, multidisciplinary management [2-4,6,8,9]. Acute gout flares are usually severely painful and highly disabling, and early involvement of rehabilitation and physiotherapy teams is important to support mobility, prevent deconditioning, and promote functional recovery [1,4,6]. Ongoing patient education on adherence to urate-lowering therapy and lifestyle modification is essential to reduce future flares and limit further disease progression [3-6,8-10].

## Conclusions

This case illustrates how chronic tophaceous gout can progress to severe, deforming polyarthritis in patients with long-standing disease and multiple comorbidities. When a substantial tophus burden coexists with cardiovascular disease, liver dysfunction, venous insufficiency, and chronic ulcers, clinicians must carefully balance infection control with the risks and benefits of anti-inflammatory therapy, glucocorticoids, and urate-lowering strategies.

The clinical course also underscores the importance of an individualized, multidisciplinary approach with a strong focus on functional recovery. Early involvement of rehabilitation teams and timely optimization of urate-lowering therapy may help preserve function and reduce the risk of future flares, even in advanced disease. Sustained patient education on adherence, medication use, and lifestyle modification is crucial to limit further disease progression and long-term functional decline.
